# Case Report: Progressive visual decline after optic neuritis associated with microcystic macular oedema

**DOI:** 10.3389/fneur.2026.1772551

**Published:** 2026-03-18

**Authors:** Federico Burguet Villena, Shaumiya Sellathurai, Kean Schoenholzer, Bettina Fischer-Barnicol, Konstantin Gugleta, Jens Kuhle, Athina Papadopoulou

**Affiliations:** 1 Department of Neurology, University Hospital Basel, Basel, Switzerland; 2 Department of Clinical Research, Faculty of Medicine, University of Basel and University Hospital Basel, Basel, Switzerland; 3 Department of Ophthalmology, University Hospital Basel, Basel, Switzerland

**Keywords:** microcystic macular oedema, MMO, ON, optic neuritis, visual decline, multiple sclerosis

## Abstract

A 39-year-old man was diagnosed with multiple sclerosis (MS) following unilateral optic neuritis (ON). His vision improved with corticosteroid treatment, but residual deficits remained (visual acuity at initial discharge: 0.7). Ocrelizumab was started, without any confirmed relapses or activity on magnetic resonance imaging (MRI) ever since. Nevertheless, his vision gradually deteriorated (0.3). Optical coherence tomography (OCT) detected microcystic macular oedema (MMO) at month 6, which showed clear progression over 5 years. MMO is rare, and longitudinal data on its evolution are scarce. In our case, a progressive MMO course was accompanied by retinal neuroaxonal thinning and gradual visual decline, despite clinical and MRI stability under ocrelizumab. This case highlights that late or progressive visual deterioration after severe ON may be associated with evolving MMO, which can be monitored by longitudinal OCT.

## Introduction

Optic neuritis (ON) is one of the most frequent clinical manifestations of multiple sclerosis (MS), while subclinical atrophy of the optic nerve and retina is also very common (1, 2). Optical coherence tomography (OCT) is a highly reproducible, non-invasive technique that can quantify both clinical (after ON) and subclinical structural retinal damage in people with MS (pwMS) (3). In addition to thinning of specific retinal layers, other, less common OCT findings have been described in MS, such as microcystic macular oedema (MMO). MMO is rare, and longitudinal data on its evolution are scarce (4).

Here, we describe a patient with relapsing–remitting MS who developed progressive MMO following acute ON, accompanied by retinal atrophy and gradual visual deterioration over 5.5 years, despite clinical and magnetic resonance imaging (MRI) stability under ocrelizumab. This case provides longitudinal insight into the evolution of MMO after ON and suggests that late or gradually worsening visual function may be associated with evolving MMO, which can be monitored by OCT.

## Case report

A 39-year-old previously healthy man was referred to our MS center by his ophthalmologists with suspected acute ON of the right eye. On admission, he reported several days of retro-orbital pressure and pain with right eye movements, most pronounced on upgaze, followed by progressively worsening blurred vision in the right eye over the preceding two days. There was no history of previous visual, sensory, or motor symptoms, and his family history was unremarkable.

Neurological examination revealed visual loss in the right eye (high-contrast visual acuity: 0.6), mild red desaturation, and a relative afferent pupillary defect (RAPD) on the right. No additional clinical abnormalities were noted. Visual evoked potentials showed conduction block after stimulation of the right eye. MRI of the brain, including an orbital protocol, showed a T2-hyperintense lesion in the right optic nerve without contrast enhancement and no other evidence of orbital pathology. Moreover, multiple FLAIR (fluid-attenuated inversion recovery) hyperintense lesions were observed in the white matter, including the corpus callosum and midbrain, none of which showed contrast enhancement.

The patient received intravenous methylprednisolone (1 g/day) for five days, which was extended to a total of eight days due to initial visual worsening (<0.1). Visual acuity subsequently improved to 0.7 at discharge, and an oral prednisone taper was initiated. Based on fulfillment of the 2017 revised McDonald criteria for dissemination in space (one juxtacortical and one infratentorial lesion) and in time (positive oligoclonal bands in the cerebrospinal fluid), the patient was diagnosed with relapsing–remitting MS. Antibody testing for MOG-IgG and aquaporin 4-IgG was not performed due to the typical MS findings and the lack of “red flags,” such as pronounced optic disc edema or a longitudinal optic nerve lesion on MRI. Two months after acute ON, he was started on ocrelizumab 600 mg every six months. At that time, the patient reported slightly worsened vision after discontinuing steroids (measured visual acuity 0.5), but no relapse treatment was administered. At month 6, there was again a mild deterioration of right-eye visual acuity without pain or acute onset (measured visual acuity 0.4), and a further course of intravenous steroids was administered, without immediate clinical improvement. Over the subsequent years, the patient was followed regularly in our department and showed fluctuating visual function around 0.5, with a gradual decline to 0.3 by year 5. There were no acute ON symptoms between month 6 and year 5, and no further relapses or MRI activity.

**Figure 1:**
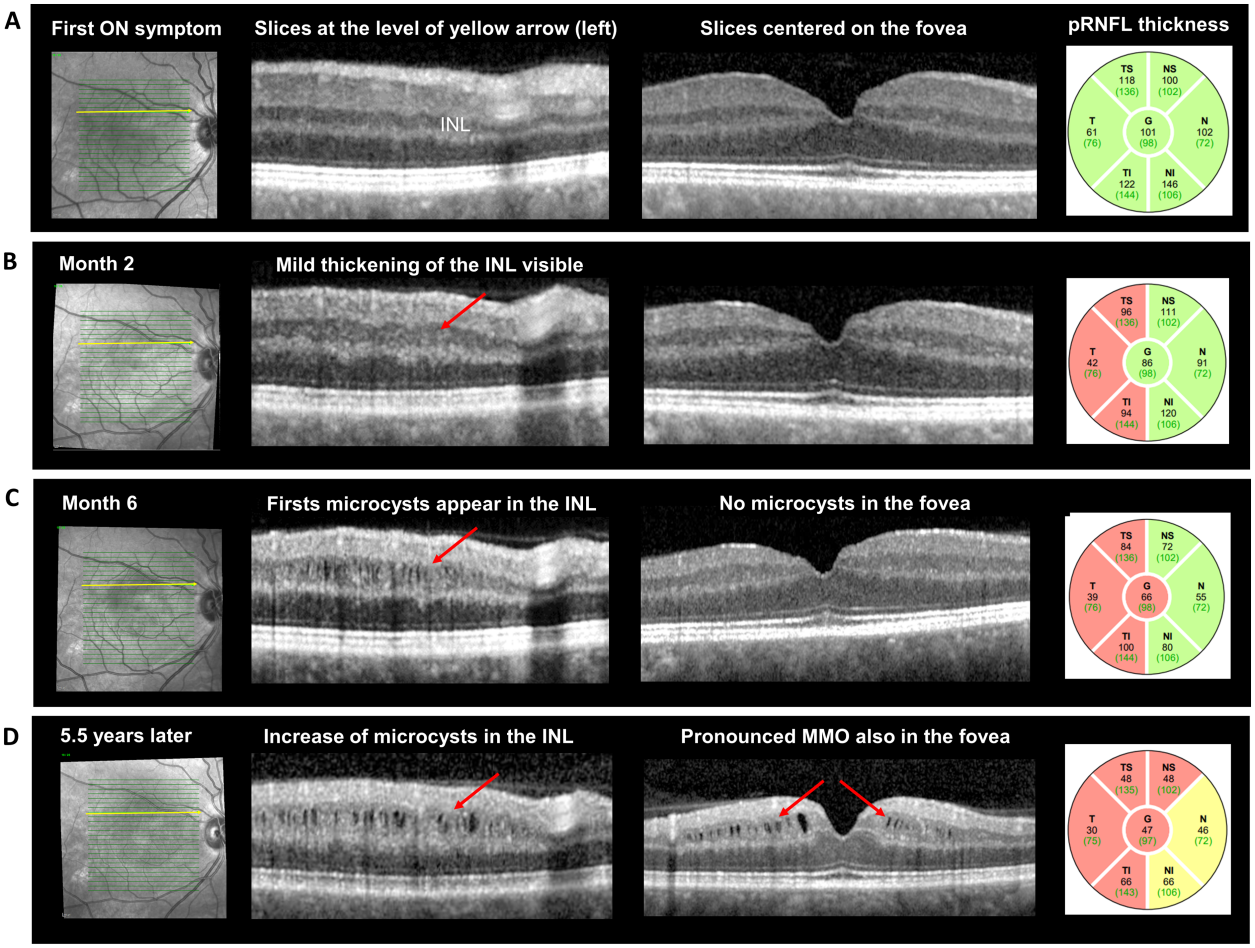
Temporal evolution of microcystic macular oedema after optic neuritis, on serial optical coherence tomography images. At time of acute ON **(A)**, OCT was normal; at month 2 **(B)** there was mild thickening of the macular INL (red arrow), without oedema, as well as atrophy of the pRNFL, pronounced on the temporal segments. At month 6 **(C)**, the first microcysts were visible in the INL, while pRNFL thickness showed a further reduction of −20 μm. After 5.5 years **(D)** the INL-cysts increased in number and size, while microcystic macular oedema was also noted in the fovea; pRNFL thinning was more pronounced and involved all the segments. pRNFL: peripapillary retinal nerve fiber layer; INL: inner nuclear layer.

Optical coherence tomography (OCT) performed 5.5 years after ON onset revealed pronounced MMO in the right inner nuclear layer of the macula (mINL; Figure 1). Retrospective review of serial OCT scans showed mild INL thickening at two months post-ON, followed by the first appearance of microcysts at month 6. Over the following years, the number, size, and extent of the cysts progressively increased toward the fovea (Figure 1). This evolution was paralleled by marked thinning of both the peripapillary retinal nerve fiber layer (pRNFL) and the macular ganglion cell–inner plexiform layers (mGCIPL). Quantitatively, the right-eye pRNFL decreased from 101 μm at baseline to 47 μm after 5.5 years, and the mGCIPL from 75.3 μm to 46.3 μm, corresponding to total thinning of −54 μm and −29 μm, respectively (Table). INL thickness in the right eye increased from 39.6 μm at baseline to 57.6 μm over the same period (Figure 1; Table 1). The left eye remained structurally and functionally stable, with a visual acuity of 1.0 and minimal change in OCT metrics (Table 1).

**Table 1:** Temporal evolution of retinal layer thicknesses in optical coherence tomography and of visual function during 5.5 years after the acute ON episode.

Time-point	OD				OS			
	pRNFL (mcm)	GCIPL (mcm)	INL (mcm)	Visual acuity	pRNFL (mcm)	GCIPL (mcm)	INL (mcm)	Visual acuity
Baseline (ON)	101	75.3	39.6	<0.1 (at discharge 0.7)	103	79.9	42	1.0
Month 2	86	54.5	42.7	0.5	103	81.3	41.7	1.0
Month 6	66	52	45.6	0.4	105	81	40.6	1.0
Month 18	—	—	—	0.5	—	—	—	1.0
Year 2	—	—	—	0.6	—	—	—	1.0
Year 3	—	—	—	0.5	—	—	—	1.0
Year 4	—	—	—	0.5	—	—	—	1.0
Year 5	47	46.3	57.6	0.3	101	78.9	40.7	1.0

Note that the macular layer thicknesses derive from the 1, 3, 6 mm ETDRS grid on macular volume scans. pRNFL, peripapillary Retinal Nerve Fiber Layer; GCIPL, Ganglion Cell-inner Plexiform Layer; ON, optic neuritis; N/A, not available.

Throughout follow-up, the patient remained neurologically stable, with an Expanded Disability Status Scale (EDSS) score ranging from 1.5 to 2.0. The higher score reflected an increase in the visual functional system score (FSS) from 1 to 3 (converted visual FSS: 2.0) due to persistent visual impairment. Laboratory tests confirmed complete B-cell depletion under ocrelizumab, and no MRI evidence of inflammatory activity was observed.

The combination of progressive visual decline, OCT-documented pronounced retinal atrophy, and worsening MMO in this patient suggests severe retrograde neurodegeneration in the retina, occurring in the absence of further relapses or MRI activity.

## Discussion

MMO can be easily identified on OCT as multiple lacunar, hyporeflective (“black”) areas, typically located in the INL. MMO is not disease-specific and has been reported in both inflammatory and non-inflammatory optic neuropathies (5). In the context of neuroinflammatory diseases, MMO can develop following (severe) ON in MS and, even more frequently, in NMOSD and MOGAD (6, 7). It should not be mistaken for cystoid macular edema, and physicians should recognize that its origin lies in the optic nerve rather than the macula itself (8). The exact mechanisms underlying MMO are unknown, but retrograde degeneration from the optic nerve to the ganglion cells—and ultimately to the bipolar cells of the macula [leading to the formation of empty spaces in the INL (9)]—seems likely.

Longitudinal data can help improve our understanding of the mechanisms underlying MMO, but such data are relatively scarce (10). In a previous case series (6), six patients were followed, showing different outcomes (improvement: *n* = 2, stable: *n* = 1, and worsening: *n* = 2). However, the temporal evolution of MMO in relation to the acute ON event was unclear. In another case series, very mild initial MMO changes were observed four months after the onset of ON in a single patient (11).

Our case showed the first appearance of microcysts between 2 and 6 months after the acute ON episode. Over longer-term follow-up (5.5 years), we observed clear worsening of MMO, accompanied by pronounced thinning of the pRNFL (−54 μm compared to baseline) and the mGCIPL (−29 μm), which corresponded to the progressive visual deficits in the right eye.

The absence of OCT scans between month 6 and year 5, as well as the lack of a specific orbital MRI protocol during follow-up, did not allow us to exactly assess the temporal dynamics of optic nerve damage in our patient. Nevertheless, the overall evolution of visual dysfunction and OCT findings over more than 5 years, in the absence of new ON episodes or other signs of clinical or MRI inflammatory activity, suggests that (retrograde) neurodegeneration was the most likely mechanism underlying MMO in our patient. Recently, the term primary progressive ON (PPON) has been used to describe a rare ON presentation characterized by progressive retinal or optic nerve atrophy and/or progressive visual loss lasting more than a year (1). The diagnosis of PPON is based on time, and a progressive (in contrast to the typical relapsing pattern) course from onset is required. In our patient, the initial ON episode was acute, with visual deterioration over a few days and initial improvement following corticosteroid treatment. However, visual decline progressed over time, corresponding to pronounced retinal atrophy and the development of MMO (Figure 1). Although the lack of serial OCT images between month 6 and year 5 is a limitation, the course observed in our patient is consistent with ongoing retrograde neurodegeneration from the optic nerve toward the retina, affecting ganglion cells, their synapses (GCIPL thinning), and bipolar cells (manifested as severe MMO in the INL). Therefore, our case illustrates progressive neurodegenerative changes in the retina, occurring in the absence of clinical or MRI signs of disease activity, in a patient with MS. Moreover, this case suggests that severe MMO can underlie late or progressive visual worsening after ON, underscoring the value of longitudinal OCT monitoring in similar patients.

## Conclusion

MMO can appear early (within 6 months) after acute ON, accompanied by pronounced thinning of the retinal axonal (pRNFL) and neuronal (mGCIPL) layers and visual deterioration. In patients with late or gradually progressive visual loss after ON, longitudinal OCT can be valuable for detecting MMO and ongoing retinal layer changes, even in the absence of other signs of disease activity or progression.

## Data Availability

The original contributions presented in the study are included in the article/supplementary material, further inquiries can be directed to the corresponding author.
